# Outcomes of Orbital Atherectomy for the Treatment of Severely Calcified Coronary Artery Lesions

**DOI:** 10.7759/cureus.37651

**Published:** 2023-04-16

**Authors:** Nouraldeen Manasrah, Mohamed Zghouzi, Ryan Naughton, Dhruvil Patel, Heba Osman, Ahmad K Abdelrahman, Adnan Halboni, Raegan Deschamps, Yasar Sattar, M. Chadi Alraies

**Affiliations:** 1 Internal Medicine, Detroit Medical Center Sinai-Grace Hospital, Detroit, USA; 2 Internal Medicine, Detroit Medical Center, Detroit, USA; 3 Internal Medicine, Wayne State University, Detroit, USA; 4 Internal Medicine, Wayne State University School of Medicine, Detroit, USA; 5 Internal Medicine/Pediatrics, Wayne State University-Detroit Medical Center, Detroit, USA; 6 Internal Medicine, Wayne State University-Detroit Medical Center, Detroit, USA; 7 Cardiology, Wayne State University-Detroit Medical Center, Detroit, USA; 8 Cardiology, West Virginia University, Morgantown, USA

**Keywords:** myocardial infarction, intravascular ultrasound (ivus), calcified coronary lesion, orbital atherectomy, cad: coronary artery disease

## Abstract

Background

Orbital atherectomy (OA) is used to prepare severely calcified coronary artery lesions before percutaneous coronary intervention (PCI). Intravascular ultrasound (IVUS) is used to determine the plaque volume and degree of stenosis within the arterial vessel. This study evaluated the safety and efficacy of OA for treating severely calcified coronary lesions and determined if IVUS impacted these outcomes.

Methods

We retrospectively collected data from a single center of patients with severe coronary artery calcification who underwent OA. The data on baseline characteristics and procedural and clinical outcomes were collected and analyzed.

Results

A total of 374 patients underwent OA. The mean age was 69 ± 12.7; 53.6% were Black, and 38% were female. Hypertension was present in 96% of the patients, followed by hyperlipidemia in 79.4%, diabetes mellitus in 53.7%, and chronic kidney disease (CKD) in 22.7%. More patients had presented with a non-ST-elevation myocardial infarction (NSTEMI) compared to ST-elevation myocardial infarction (STEMI) at 36.3% versus 4.3%, respectively. The radial artery was used in 35.4% of the cases, and the left anterior descending artery (LAD) was the most commonly treated vessel with OA at 61%, followed by the right coronary artery (RCA) at 30.7%. IVUS was utilized in 63.4% of cases. The most common complication of the procedure was perforation and dissection at an equal proportion of 1.3% among all patients. The no-reflow rate was 0.5%, and 0.5% developed post-procedural myocardial infarction (MI). The average length of stay was 4.7 days, while a marginal proportion, at 10.5%, had same-day discharge with no recorded complications.

Conclusion

In this analysis of patients with severely calcified coronary lesions, OA had low rates of major adverse cardiovascular events (MACE) and was considered a safe and effective treatment for complex coronary lesions.

## Introduction

Coronary artery calcification (CAC) score is commonly measured using cardiac computed tomography (CT) to determine the degree of atherosclerosis within coronary vessels. CAC is independently associated with age, male gender, hypertension, diabetes, hypercholesterolemia, and obesity [1]. The presence of severe CAC causes a reduction of vascular compliance and impairment of myocardial perfusion [2], which leads to a higher myocardial infarction (MI) and mortality rate [3]. It also has a negative impact on the success of percutaneous coronary intervention (PCI) [4].

Orbital atherectomy (OA) is a supplementary tool used during PCI to modify calcified coronary lesions to facilitate proper balloon and stent expansion [5]. According to the American Heart Association, an estimated 20.1 million Americans over the age of 20 have coronary artery disease, and CAC is prevalent in 32.0% of females and 52.9% of males between the ages of 45 and 75 years [6].

OA has been previously shown as a safe and effective method to improve the compliance of calcified lesions to reduce procedural complications and facilitate stent implantation [7]. The 2021 ACC/AHA/SCAI (American College of Cardiology/American Heart Association/Society for Cardiovascular Angiography and Interventions) Guidelines for Coronary Artery Revascularization currently lists the use of OA for plaque modification on fibrotic or heavily calcified lesions as a class 2b recommendation [8]. We aimed to evaluate OA's safety and efficacy for treating severely calcified coronary artery lesions within our patient population.

## Materials and methods

We retrospectively identified the patients with severe CAC who underwent OA in a single hospital system. First, the baseline demographics and comorbidities were collected from the hospital chart review, such as age, gender, race, smoking status, hypertension, diabetes, and dyslipidemia. Also, we gathered data on patient presentations, including whether patients presented with STEMI, NSTEMI, or angina, and the average troponin levels. Finally, procedural characteristics like lesion stenosis rate, balloon dilation before atherectomy, real-time imaging, minutes of average fluoroscopy time, and access site were collected and analyzed.

The study's primary objectives were to determine the most commonly involved target vessel, the average length of hospital stay (LOS), and the incidence of procedural complications, including perforation, dissection, no-reflow rates after the intervention, and post-procedural MI and stroke. All data on baseline comorbidities, procedural outcomes, and clinical outcomes were analyzed using Microsoft Excel (Microsoft® Corp., Redmond, WA).

## Results

A total of 374 patients were included in this investigation, among which 160 (42.8%) cases were treated as outpatients. The average age of the patient population was 69.3 years, and 38% were female. The most common comorbidity was hypertension (96%), followed by hyperlipidemia (79.4%) and diabetes mellitus (53.7%). Furthermore, the history of chronic kidney disease (CKD) comprised 22.7% of the patients, among which 17.4% had end-stage renal disease. The average ejection fraction in the population was 42.0 + 7.4% (Table 1).

**Table 1:** Baseline characteristics of patients undergoing orbital atherectomy (N = 374) Values are expressed as mean ± SD for continuous variables or percentages for categorical variables.

Baseline characteristics	
Age (years)	69.3 ± 9.17
Female gender	38.0%
Race	
White	29.9%
Black	53.6%
Asian	0.5%
Other	15.9%
Elective hospitalization	42.8%
Clinical presentation	
ST-elevation myocardial infarction (STEMI)	4.3%
Non-ST-elevation myocardial infarction (NSTEMI)	36.3%
Unstable angina	49.3%
Stable angina	12.7%
Troponin (ng/L)	1603 ± 4000
Positive stress test	30.6%
Comorbidities	
Hypertension	96.0%
Diabetes mellitus	53.7%
Hyperlipidemia	79.4%
Chronic kidney disease	22.7%
Mean creatinine value	2.0 ± 2.2
End-stage renal disease	17.4%
Peripheral artery disease	18.2%
Stroke	9.4%
Chronic obstructive pulmonary disease (COPD)	16.6%
Coronary artery disease	78.3%
Heart failure	41.7%
Mean ejection fraction	42.0 ± 17.4%
Prior coronary artery bypass grafting	14.2%
Smoking	58.4%

More patients presented with an NSTEMI as compared to a STEMI at 36.3% versus 4.3%, respectively, with average troponin of 1,603 + 4,000 ng/L. More patients were intervened through femoral access as compared to radial access (64.6% versus 35.4%). The most common lesion that intervened was the LAD (61.0%), while the second most common was the RCA (30.7%) (Table 2).

**Table 2:** Characteristics of orbital atherectomy procedure (N = 374) Values are expressed as mean ± SD for continuous variables or percentages for categorical variables.

Arterial Access	
Radial	35.4%
Femoral	64.6%
Target vessel (multiple vessels may be targeted in a single atherectomy procedure)	
Left anterior descending (LAD)	61.0%
Left circumflex (LCX)	25.1%
Left main (LM)	16.0%
Right coronary artery (RCA)	30.7%
Ramus intermedius (RI)	1.6%
Lesions	
Mean primary lesion length (mm)	29.2 ± 13.0
Mean primary lesion stenosis	85.0 ± 9.2%
Mean secondary lesion length (mm, N = 170)	27.7 ± 11.8
Mean secondary lesion stenosis (mm, N = 170)	84.3 ± 10.4%
Mean tertiary lesion length (mm, N = 58)	27.3 ± 10.5
Mean tertiary lesion stenosis (mm, N = 58)	84.4 ± 10.6%
Stents	
Mean number of stents used	1.7 ± 0.9
Mean first stent length (mm)	28.9 ± 9.2
Mean first stent diameter (mm)	3.2 ± 1.2
Mean second stent length (mm, N = 171)	26.5 ± 9.3
Mean second stent diameter (mm, N = 171)	3.2 ± 0.6
Mean third stent length (mm, N = 54)	27.3 ± 10.6
Mean third stent diameter (mm, N = 54)	3.1 ± 0.5
Balloon	
Balloon dilation prior to atherectomy	97.7%
Mean balloon diameter (mm)	2.7 ± 0.6
Mean balloon length (mm)	20.6 ± 7.5
Procedure	
Intravascular ultrasound (IVUS) used	63.4%
Mean number of CSI passes	2.4 ± 2.5
Mean total fluoroscopy time (min)	33.9 ± 23.5
Mean contrast volume used (mL)	186.4 ± 78.4

The average LOS for all patients was 4.7 days, while a marginal proportion of 10.5% had same-day discharge with no recorded complications (Table 3).

**Table 3:** In-hospital outcomes for orbital atherectomy procedure (N = 374) Values are expressed as mean ± SD for continuous variables or percentages for categorical variables.

In-hospital outcomes	
Mean length of stay (days)	4.7 ± 7.0
Same-day discharge	10.5%
Adverse outcomes	
Post-procedure myocardial infarction	0.5%
Vessel perforation	1.3%
Vessel dissection	1.3%
Vessel thrombosis	0.3%
Post-procedure stroke	0.8%
No reflow	0.5%

The most common complication for the procedure was perforation and dissection at an equal proportion of 1.3% among all patients, while no-reflow was evidenced in 0.5% of patients. Post-procedural MI was 0.5% of total cases. No-reflow rates after intervention were also captured to determine the effectiveness of OA clearance, which was very small at 0.5% of all total interventions. Compared to the brevity of cases, the occurrence of these outcomes was not considered in relation to the number of interventions (Table 3).

In investigating additional procedural characteristics, among 170 patients, 85.0 ± 9.2% was the average lesion stenosis rate for the primary lesion (84.3 ± 10.4%) alongside a subsequent secondary lesion, while the average stenosis was 84.4 ± 10.6% among 58 patients who were intervened with tertiary care. On average, 1.7 + 0.9 stents were used in all the procedures. In preparation for intervention, 97.7% of lesions were ballooned prior to intervention at a mean diameter of 2.7 + 0.6 mm. Most procedures also required real-time imaging, via intravascular ultrasound, for effective imaging at 63.4% of total interventions recorded in this study. At 33.9 + 23.5 minutes of average fluoroscopy time with 186.4 + 78.4 mL of contrast used, there was wide variability in the procedure time (Table 2).

## Discussion

Several methods of PCI have been used in patients with CAC, including balloon angioplasty, cutting balloons, atherectomy, stenting, and laser. Atherectomy provides higher procedural success rates for such lesions than no atherectomy [9]. Despite that, it has only been used in less than 5% of PCIs in patients with CAC [10]. The currently available coronary atherectomy devices include OA (Diamondback 360), rotational atherectomy (RA), and laser atherectomy (ELCA™).

OA is a mounted diamond-coated crown that exerts a centrifugal force to orbit, improving calcified lesions' compliance, reducing procedural complications, and facilitating stent implantation. It is the only atherectomy device approved by the US Food and Drug Administration (FDA) for treating severely calcified coronary lesions [10]. The ORBIT II trial confirmed the safety and efficacy of the coronary orbital atherectomy system (OAS) to facilitate stent placement in severely calcified coronary lesions. It reported a high rate of successful stent placement (97.7%) with 89.6% freedom from MACE, defined to be acute MI, stroke, perforation, dissection, or thrombus, in 30 days compared to the performance goal of 83 with a total perforation rate of 1.8% [11].

In this single-center retrospective analysis, we investigated the outcomes of patients who underwent OA before PCI. Post-procedure complications were relatively minimal, with post-procedural MI and stroke being recorded at 0.5% and 0.8%, respectively. Other studies into the sequelae of OA use have defined similar trends [12,13] similar to this study's patient population.

Our results demonstrated that a marginal proportion of the patient population had vascular wall injury, including perforation and dissection, at an equal ratio of 1.3% among all patients. While studies comparing RA to OA have concluded that OA has a greater proportion of coronary artery dissections and perforations [14], our results have shown that this incidence remains marginally equal relative to rates presented in studies on OA use as a part of its evaluation [15-17].

Most vessels that were intervened in our study were the LAD at 61.0% of total cases, followed by the RCA. As OA requires the need for 6Fr guides, using the high-speed feature is preferred in treating larger diameter vessels without the need to upsize the guide catheter; it also creates more calcium modification in lesions with larger lumen compared to the standard RA use [14,18]. Most of the patients undergoing intervention in our study had femoral access due to the increasing number of lesion interventions required, while approximately one-third needed radial access.

Our study includes high-risk patients, with more than half having concurrent comorbidities, including diabetes, more than two-thirds with hyperlipidemia, and almost a fifth having CKD. These pathologies have been shown to reduce the patency and decrease compliance of coronary vessels, which can contribute to worsening outcomes, especially with mechanical stress from device implementation. The design of the OA tip includes a crown that localizes the region of effect and allows for both bidirectional motions, thereby reducing the radiance of thermal injury and producing smaller particulates [19].

No reflow is also noted as a rare complication of OA use [11] that could result from distal thrombosis during the procedure and ineffective plaque clearance as evaluated by intravascular ultrasound [7]. Lastly, the incidence of cardiac tamponade, most likely a sequela from coronary dissection or rupture, was not assessed in this study but was noted in other OA evaluations to be extremely minimal [12,17].

## Conclusions

Severely calcified coronary lesions are a type of complex coronary lesions, which are challenging to treat using conventional techniques. OA is a viable and effective treatment option for severely calcified coronary lesions, with minimal post-procedural complications. However, further research is needed to confirm its effectiveness and safety and to explore its potential benefits in a broader range of patients and conditions.
